# TDG suppresses the migration and invasion of human colon cancer cells via the DNMT3A/TIMP2 axis

**DOI:** 10.7150/ijbs.69266

**Published:** 2022-03-21

**Authors:** Jiyu Miao, Changan Zhao, Kaijie Tang, Xiaofan Xiong, Fei Wu, Wanjuan Xue, Baojun Duan, Huahua Zhang, Xintao Jing, Wen Li, Ying Sun, Ni Hou, Chen Huang

**Affiliations:** 1 Department of Cell Biology and Genetics, School of Basic Medical Sciences, Xi'an Jiaotong University Health Science Center, Xi'an 710061, China.; 2 Department of Hematology, The Second Affiliated Hospital of Xian Jiaotong University, Xi'an 710004, China.; 3 Department of Pathology, School of Basic Medical Sciences, Xi'an Jiaotong University Health Science Center, Xi'an 710061, China.; 4 Institute of Genetics and Developmental Biology, Xi'an Jiaotong University, Xi'an 710061, China.; 5 National Local Joint Engineering Research Center of Biodiagnostics and Biotherapy, The Second Affiliated Hospital of Xian Jiaotong University, Xi'an 710004, China.; 6 Department of Oncology, The Second Affiliated Hospital of Xi'an Jiaotong University, Xi'an, 710004, China.; 7 State Key Laboratory of Military Stomatology & National Clinical Research Center for Oral Diseases & Shaanxi Key Laboratory of Stomatology, Department of oral biology, School of Stomatology, The Fourth Military Medical University, Xi'an 710032, China.; 8 Department of Medical Oncology of Shaanxi Provincial People's Hospital, Xi'an 710068, China.; 9 Medical Research and Experimental Center, Medical College, Yan'an University, Yan'an 716000, China.; 10 Key Laboratory of Environment and Genes Related to Diseases, Ministry of Education of China, Xi'an Jiaotong University, Xi'an 710061, China.; 11 Key Laboratory of Shaanxi Province for Craniofacial Precision Medicine Research, Xi'an 710004, China.

**Keywords:** Colorectal cancer, TDG, DNMT3A, TIMP2, metastases, methylation

## Abstract

**Background:** Colorectal cancer (CRC) is one of the most common malignant tumors with high rates of recurrence and mortality. Thymine DNA glycosylase (TDG) is a key molecule in the base excision repair pathway. Recently, increasing attention has been paid to the role of TDG in tumor development. However, the specific functions of TDG in CRC remain unclear.

**Methods:** The biological functions of TDG and DNA methyltransferase 3 alpha (DNMT3A) in CRC were evaluated using migration and invasion assays, respectively. A tumor metastasis assay was performed in nude mice to determine the *in vivo* role of TDG. The interaction between TDG and DNMT3A was determined via co-immunoprecipitation (Co-IP). Chromatin immunoprecipitation analysis (ChIP) was used to predict the DNA-binding site of DNMT3A. We also performed methylation-specific PCR (MSP) to detect changes in TIMP2 methylation.

**Results:** TDG inhibited the migration and invasion of human colon cancer cells both* in vitro* and *in vivo*. TDG promoted the ubiquitination and degradation of DNMT3A by binding to it. Its interference with siDNMT3A also inhibits the migration and invasion of human colon cancer cells. Furthermore, the ChIP, MSP, and rescue experiments results confirmed that TDG accelerated the degradation of DNMT3A and significantly regulated the transcription and expression of TIMP2, thereby affecting the migration and invasion of human colon cancer cells.

**Conclusion:** Our findings reveal that TDG inhibits the migration and invasion of human colon cancer cells through the DNMT3A-TIMP2 axis, which may be a potential therapeutic strategy for the development and treatment of CRC.

## Introduction

Colorectal cancer (CRC) is one of the most common malignant tumors that seriously threatens human health 1. CRC development is a complex multi-step process that is induced by various environmental and genetic factors, such as gene mutations and epigenetic modifications. The current treatment for patients with CRC is still based on surgery and is supplemented by radiotherapy and chemotherapy 2. However, the 5-year survival rate of patients with CRC is relatively low and the prognosis is not ideal. The main reason for this is the high metastasis and recurrence rates of CRC 3.

Thymine DNA glycosylase (TDG) is a key molecule in the base excision repair (BER) pathway that removes thymine from a G/T mispair and restores a G/C base pair following 5-methylcytosine deamination 4. With the progress of research, other functions of TDG have emerged, particularly its involvement in epigenetic modifications, such as DNA demethylation 5, 6. TDG can recognize various derivatives of 5-methylcytosine and remove them to achieve demethylation. TDG also interacts with DNA methyltransferase 3 alpha (DNMT3A) to inhibit its methylation activity 7. As a methyltransferase, DNMT3A performs the catalytic function of directly methylating the CpG island of the target gene promoter and changing its methylation level to achieve a transcriptional silencing effect 8, 9. Cells with DNA repair defects have increased genomic instability and are more likely to acquire mutations that lead to cell transformation and cancer, such as Lynch syndrome tumors and *MUTYH*-associated polyposis syndrome (MAP) 10. However, current research on TDG mainly focuses on the gene mutation caused by its abnormality, and research on its role and mechanism in carcinogenesis and cancer development is still very limited; this needs to be explored for specific cancers.

In the present study, we analyzed the significance of TDG in CRC using publicly available CRC gene expression RNA sequencing (RNA-seq) datasets from The Cancer Genome Atlas (TCGA). TDG is closely associated with CRC metastasis. *In vitro* and *in vivo* experiments were performed to examine the role of TDG in CRC. The TDG-DNMT3A interaction and its function in the role of TDG in CRC were explored in depth. Our results strongly demonstrate the epigenetic regulatory role of TDG in inhibiting CRC metastasis and will enrich the knowledge about the regulatory mechanisms of TDG and its potential role in therapeutic strategies for CRC.

## Materials and methods

### Bioinformatics analysis

TCGA database and a designed web tool (https://xenabrowser.net/) were used to investigate the relative expression of TDG and its correlation with the CRC stage and prognosis. We used the UCSC Genome Browser (http://genome.ucsc.edu/) to download the 2,000 bp DNA sequence upstream of the *TIMP2* transcription initiation start site as a *TIMP2* promoter. Then, Primer 5.0 software was used to design the PCR primers for ChIP. We also used MethPrimer 2.0 (http://www.urogene.org/methprimer2/) to predict the methylation sites of the *TIMP2* promoter and design the PCR primers for MSP.

### Clinical patient samples

Colorectal cancer samples were collected from patients at the Second Affiliated Hospital of Xi'an Jiaotong University. All results were validated via pathological examination. This study was approved by the Research Ethics Committee of the Xi'an Jiaotong University, and all patients provided their written informed consent. The tissue samples were immediately frozen in liquid nitrogen until protein extraction.

### Cell lines and cell culture

Human colon cancer cells (HCT116, RKO, SW480, and SW620) and the normal colonic epithelial cell line NCM460 were purchased from the American Type Culture Collection. Cells were cultured in RPMI-1640 medium supplemented with 10% (v/v) fetal bovine serum (Gibco BRL, New York, NY, USA) at 37 °C, 95% air, and 5% CO2 in a humidified atmosphere.

### Quantitative real-time PCR

Total RNA was extracted using the TRIzol reagent (Invitrogen Life Technologies, USA). cDNA was generated using a StarScript II First-strand cDNA Synthesis Mix Kit (GenStar, Beijing, China). Quantitative real-time PCR (qRT-PCR) was performed using an FTC-3000^TM^ System (Funglyn Biotech Inc., Canada). The relative gene expression was calculated using the 2^-ΔΔCt^ method.

### Migration and invasion assay

Transfected HCT116 and SW480 cells were resuspended in 200 μL of serum-free RPMI-1640 medium and plated in the upper chamber of a Transwell coated with or without Matrigel. The lower chamber was filled with RPMI-1640 complete medium. After incubation for 24 and 36 h, the cells on the bottom surface were fixed with 4% paraformaldehyde and stained with 0.1% crystal violet. Images were captured using an inverted microscope. Then, 500 μL of glacial acetic acid was added and the mixture was shaken for 30 min to dissolve the crystal violet. The solution was transferred to a microplate (100 μL per well) and the absorbance was measured at 570 nm using a spectrophotometer (FLUOstar OPTIMA, BMG, Germany).

### Western blotting

Cells were lysed using RIPA buffer (Pioneer, Shanghai, China) containing phenyl methyl sulfonyl fluoride (Sigma, Germany). A BCA protein assay kit (GenStar, Beijing, China) was used to measure the protein concentrations. Primary antibodies (1:1,000) (Proteintech, Wuhan, China) were dropped onto the membranes and incubated overnight at 4 °C. All membranes were then incubated with a secondary antibody (Abways Technology, China) for 1 h at room temperature. The protein expression for each sample was normalized to glyceraldehyde-3-phosphate dehydrogenase.

### Chromatin immunoprecipitation analysis (ChIP)

ChIP detects DNA fragments bound to specific proteins via co-precipitation with chromatin and polymerase chain reaction (PCR). The DNA and proteins were cross-linked and ultrasonically broken into small fragments. The DNA fragments were captured using the DNMT3A antibody and purified for PCR using three pairs of ChIP-specific TIMP2 primers (Table S1). The products were separated via agarose gel electrophoresis. A gel imaging system was used to obtain images.

### Methylation-specific PCR (MSP) analysis

After treating the DNA with sodium bisulfite, all the unmethylated cytosines are deaminated into uracil, whereas the methylated cytosines remain unchanged. Based on this base change, the primers for the methylated and unmethylated sequences were designed and used to amplify the sequences via PCR. Finally, the methylation status of the DNA sequences complementary to the primers was determined using agarose gel electrophoresis. An EpiTect Bisufite Kit (QIAGEN, Germany) was used for sulfite conversion. All operations were performed according to the manufacturer's instructions. Four pairs of methylated and unmethylated TIMP2 primers are listed in Table S1.

### *In vivo* Tumor Metastasis Assay

4-6-week-old male nude mice were purchased and raised in a pathogen-free SPF room at the Animal Center of Xi'an Jiaotong University. All tumorigenic experiments were conducted under the guidance of the Animal Health and Utilization Committee of the Xi'an Jiaotong University. 100 µL (5 × 10^6^ cells) of HCT116 Lv-ctrl or HCT116 Lv-TDG stably transfected cell suspension was injected into the mice through the tail vein. Then, the mice were raised for 3 weeks and intraperitoneally injected with 100 µL of a D-fluorescein sodium salt solution (15 mg/mL), and lung metastasis was monitored using an IVIS bioluminescence imaging system.

### Statistical analysis

Data are presented as the mean ± SD of, at least, three independent experiments. Student's *t*-test was used to analyze the differences between the groups. Statistical significance was set at* p* < 0.05. All statistical analyses were performed using IBM SPSS Statistics 23 software.

## Results

### TDG is downregulated in the mCRC samples and CRC cells

**Figure 1:**
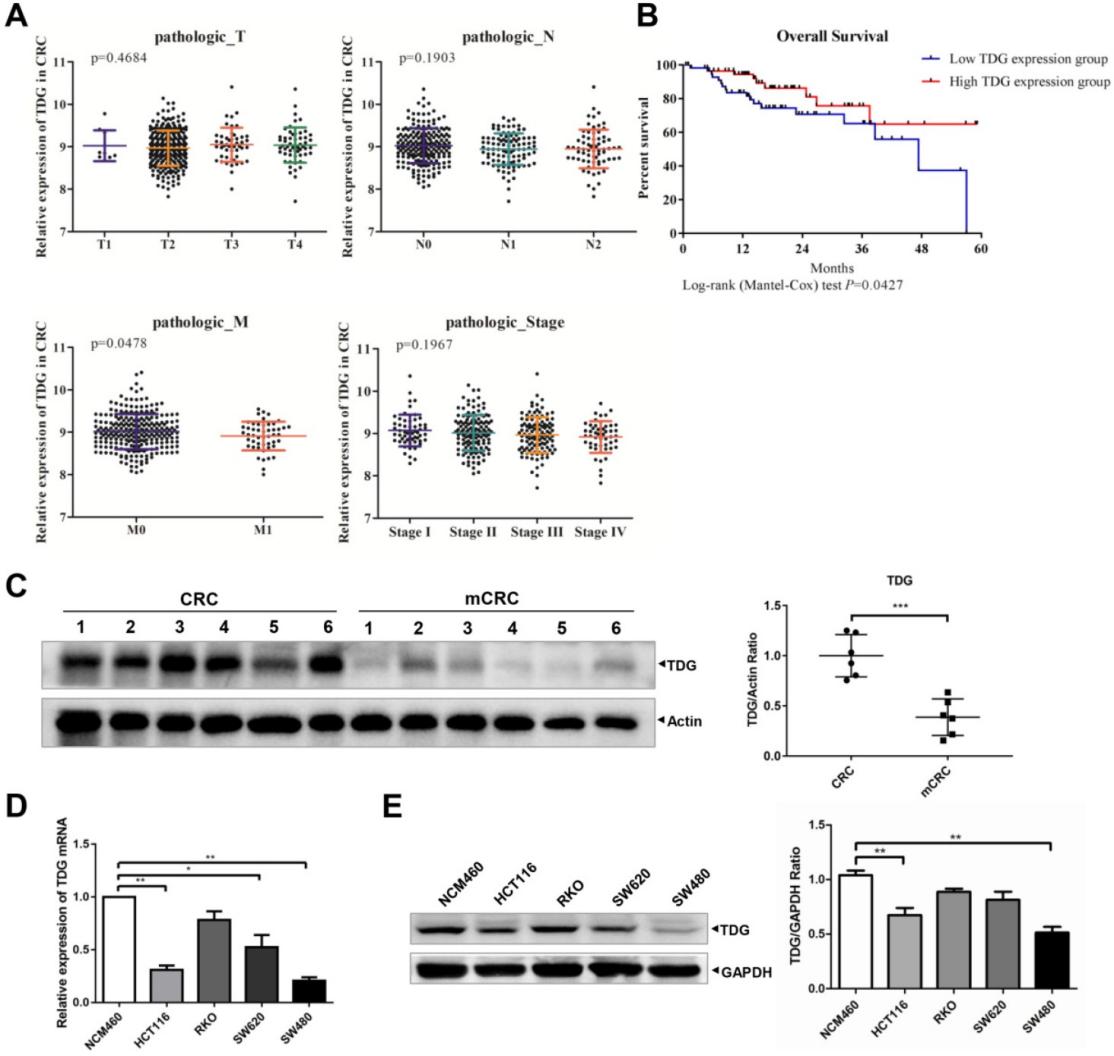
**Bioinformatics analysis and TDG expression in CRC patient tissues and cells. (A)** TDG expression in different pathologic classification of CRC patients.** (B)** Low TDG expression related to poor prognosis. **(C)** The expression of TDG was decreased in mCRC (metastasis CRC) compared with CRC. **(D-E)** The expression of TDG in NCM460 and CRC cells were detected by qRT-PCR and western blotting. *P<0.05, **P<0.01, ***P<0.001.

To explore the role of TDG in CRC, we analyzed the relationship between TDG expression and the TNM and stage of the patients with CRC through their samples using publicly available CRC gene expression RNA-seq data from the TCGA database. The relationship between TDG expression and the overall survival of patients with CRC was also analyzed. TDG expression was not associated with tumor topography (T), lymphatic metastasis (N), or the stage of patients but was significantly correlated with distant metastasis (M). TDG expression was significantly decreased in patients with metastatic CRC (M1) (Figure 1A). In addition, a low TDG expression was associated with a poor overall survival in patients with CRC (Figure 1B). We further examined the TDG expression in the CRC tissues and in human colon cancer cells. Compared with that in the CRC tissues without metastasis, the TDG protein was remarkably downregulated in the metastatic CRC tissues (mCRC) (Figure 1C). Lower TDG mRNA (Figure 1D) and protein expression (Figure 1E) were also detected in human CRC cells compared with those in the normal human colonic epithelial cell line, NCM460. These results indicate that TDG is downregulated in mCRC samples and in human CRC cells and is associated with CRC metastasis and prognosis in patients with CRC.

### TDG inhibits CRC metastasis *in vitro* and *in vivo*

**Figure 2:**
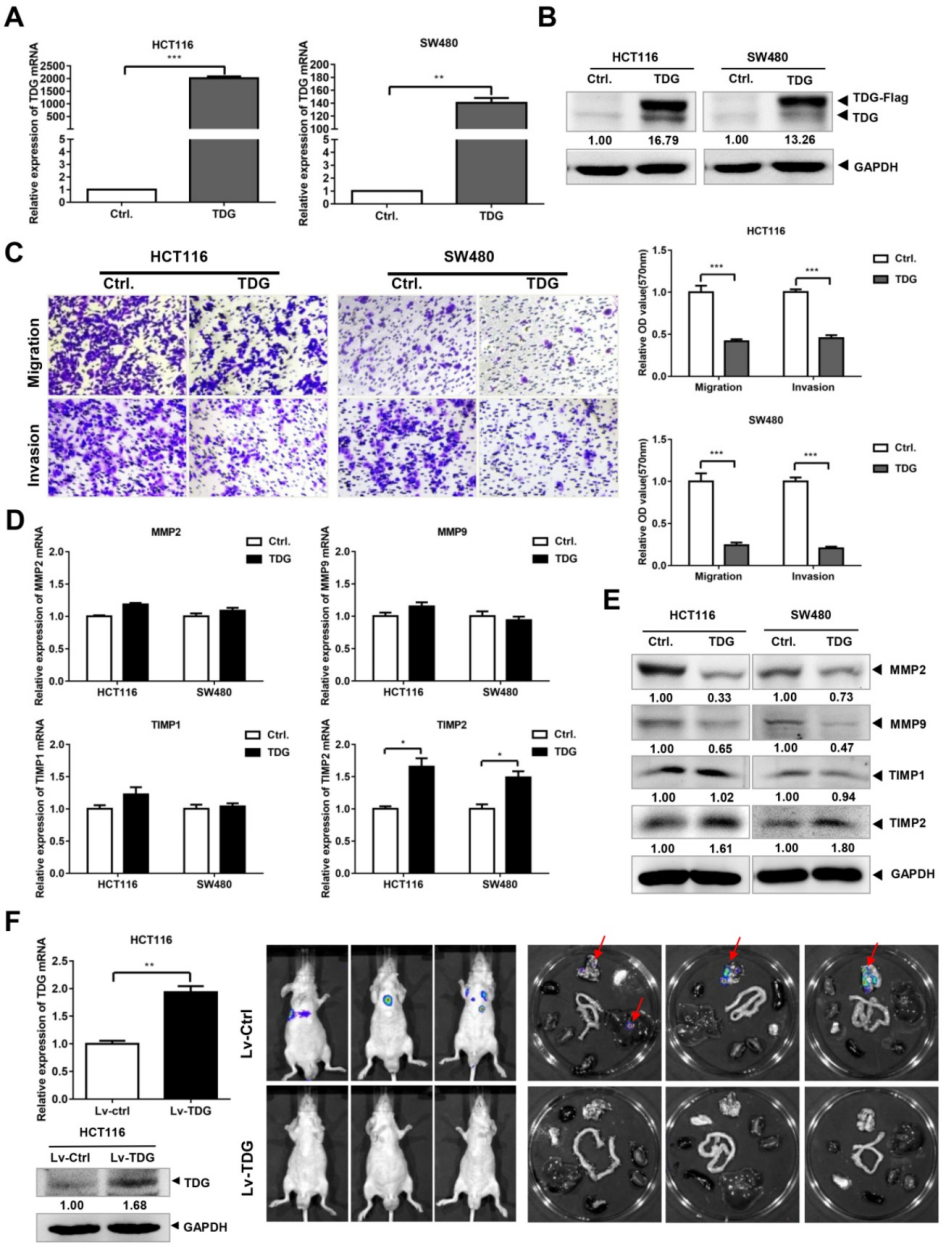
**TDG inhibited the migration, invasion and metastasis of colon cancer cells. (A-B)** The overexpression efficiency of TDG was detected by qRT-PCR and western blotting. **(C)** The transwell assay was performed for migration and invasion of colon cancer cells. **(D-E)** Migration and invasion related molecules were detected by qRT-PCR and western blotting. F. Construction of metastatic tumor model in nude mice. *P<0.05, **P<0.01, ***P<0.001.

To explore its role in CRC, we constructed a TDG overexpression vector with a FLAG tag. This vector and a control vector were transfected into two human CRC cell lines: HCT116 and SW480. qRT-PCR and western blotting were performed to verify the TDG overexpression. The results showed that compared with those of the control (Ctrl.), the mRNA (Figure 2A) and protein levels (Figure 2B) of TDG in the TDG overexpression group (TDG) were significantly increased in both cell lines. A Transwell assay was used to detect the effect of TDG on the migration and invasion of CRC cells (Figure 2C). The results showed that the number of HCT116 and SW480 cells that passed through the chamber with or without Matrigel was significantly reduced in the TDG group compared with that of the control, indicating a decline in cell migration and invasion induced by TDG. We then detected the molecular changes related to tumor migration and invasion. The qRT-PCR results showed that there was no significant difference in the mRNA levels of MMP2, MMP9, and TIMP1 between the TDG and control groups. However, the mRNA level of TIMP2 increased significantly in the TDG group compared to that in the control group (Figure 2D). The western blotting data also showed that the protein level of TIMP2 increased significantly in the TDG group, whereas no changes in the TIMP1 protein level was observed. Moreover, a decrease in the MMP2 and MMP9 protein levels in the TDG group compared to that of the control group was observed (Figure 2E). These results indicate that TDG significantly inhibited the migration and invasion ability of CRC cells.

We then established a mouse metastasis model to explore the effect of TDG on the metastasis of CRC cells *in vivo*. Lentiviruses of TDG and the control were constructed and transfected into HCT116 cells to create stable overexpressing cells, HCT116-Lv-TDG, and control cells, HCT116-Lv-Ctrl. These were injected into the tail veins of nude mice and, 3 weeks later, metastasis imaging was performed using a small-animal live imaging system. There were metastatic sites in the lungs of the nude mice in the HCT116-Lv-Ctrl group, but no metastases were observed in the HCT116-Lv-TDG group (Figure 2F); that is, TDG overexpression inhibited the metastasis of human colon cancer cells *in vivo*. These data are consistent with the result of the TDG-mediated inhibition of the migration and invasion of colon cancer cells.

### TDG could bind to DNMT3A and promote the ubiquitination and degradation of DNMT3A

**Figure 3:**
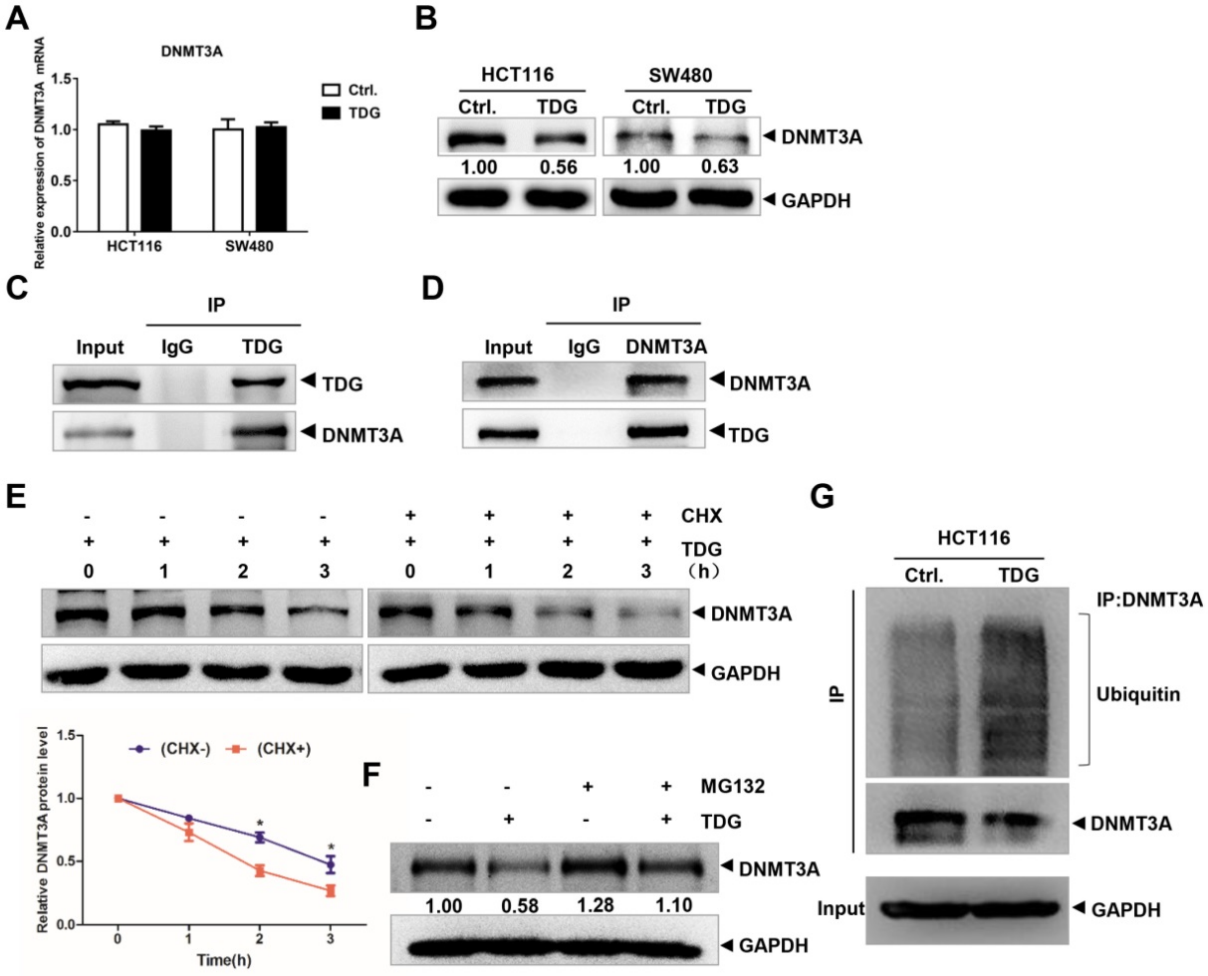
** TDG interacts with DNMT3A. (A-B)** The expression of DNMT3A was detected by qRT-PCR and western blotting after transfected with TDG plasmid. **(C-D)** Co-IP was applied to vivificate the combination of TDG and DNMT3A. **(E)** CHX promoted the degradation rate of DNMT3A. **(F)** MG132 inhibited the decrease of DNMT3A caused by TDG.** (G)** TDG increased the ubiquitination level of DNMT3A. *P<0.05, **P<0.01, ***P<0.001.

Previous studies have reported that TDG plays an important role in the epigenetic regulation of gene demethylation by interacting with DNMT3A. Therefore, we focused our research on the interaction between TDG and DNMT3A in human CRC cells. We transfected the TDG overexpression vector and the control vector into HCT116 and SW480 cells, respectively, and examined the changes in DNMT3A expression using qRT-PCR and western blotting. The results showed that there was no significant difference in the mRNA level of DNMT3A between the two groups (Figure 3A); however, the protein level of DNMT3A in the TDG overexpression group was significantly lower than that in the control group (Figure 3B). The expression of DNMT3A was higher in the mCRC tissues than in the non-metastatic CRC tissues (Supplementary Figure 1A-B). The Co-IP assay results confirmed that TDG could bind to DNMT3A in HCT116 cells (Figure 3C-D). Thus, there is an interaction between TDG and DNMT3A, and TDG can downregulate the expression of DNMT3A post-translationally in human CRC cells.

To further explore the mechanism by which TDG regulates the expression of DNMT3A, we added imine cyclohexanone (CHX), a protein synthesis inhibitor, to HCT116 cells in the TDG group to a concentration of 1 μM, extracted the protein lysates after 0, 1, 2, and 3 h, and performed western blotting. The results showed that the addition of CHX caused the TDG-induced DNMT3A protein level to decline significantly faster than in the control group (Figure 3E). Moreover, MG132, a proteasome inhibitor, was added to the HCT116 cells of the Ctrl. and the TDG groups, respectively, to a concentration of 1 μM. After 24 h, the protein lysates were extracted and subjected to western blotting. The results showed that MG132 inhibited the TDG-induced downregulation of the DNMT3A protein (Figure 3F). These data suggest that TDG might be involved in the proteasomal degradation of the DNMT3A protein. We then performed a Co-IP experiment to capture the DNMT3A protein and its interactors, and detected the amount of these proteins that were ubiquitinated (Figure 3G). The ubiquitination of the DNMT3A protein in the TDG group increased significantly compared to that in the control group. Therefore, we propose that TDG binds to DNMT3A and promotes its ubiquitination and degradation.

### The knockdown of DNMT3A suppresses the migration and invasion of CRC cells

**Figure 4:**
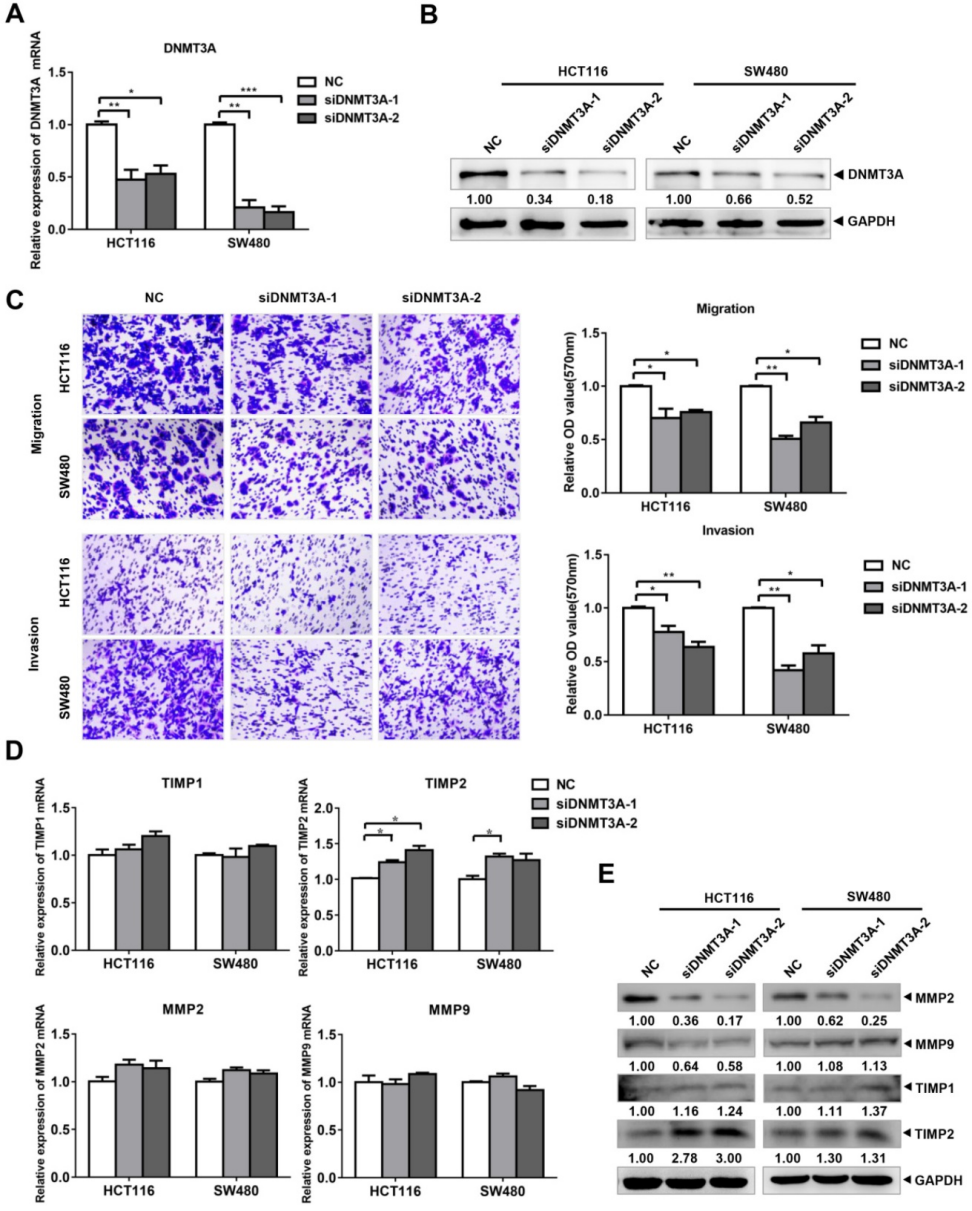
**siDNMT3A suppressed the migration and invasion of colon cancer cells. (A-B)** The inhibition efficiency of DNMT3A was detected by qRT-PCR and western blotting. **(C)** The transwell assay was performed for migration and invasion of colon cancer cells.** (D-E)** Migration and invasion related molecules were detected by qRT-PCR and western blotting after transfected with siDNMT3A-1/2. *P<0.05, **P<0.01, ***P<0.001.

To verify the role of DNMT3A in the migration and invasion of CRC cells, we designed and purchased DNMT3A interference fragments and transfected them into HCT116 and SW480 cells. The results of qRT-PCR (Figure 4A) and western blotting (Figure 4B) verified the knockdown of both DNMT3A mRNA and protein in the cells of the siDNMT3A group (siDNMT3A-1, siDNMT3A-2). Transwell assays were then used to detect the changes in the migration and invasion of these two cell lines (Figure 4C). The results showed that compared with those of the control group (NC), the migration and invasion abilities of HCT116 and SW480 cells in the siDNMT3A group were decreased. The qRT-PCR results showed that there were no significant differences in the mRNA levels of MMP2, MMP9, and TIMP1 between the siDNMT3A group and the NC (Figure 4D). However, the mRNA level of TIMP2 in the siDNMT3A group was significantly higher than that in the NC. The western blotting results also showed that the protein level of TIMP2 in the siDNMT3A group was significantly higher than that in the control group, with no significant changes in the TIMP1 protein level and a decrease in the MMP2 and MMP9 protein levels in the siDNMT3A group (Figure 4E). Therefore, DNMT3A knockdown suppresses the migration and invasion abilities of human CRC cells.

### DNMT3A binds to the TIMP2 promoter to regulate its methylation level

**Figure 5:**
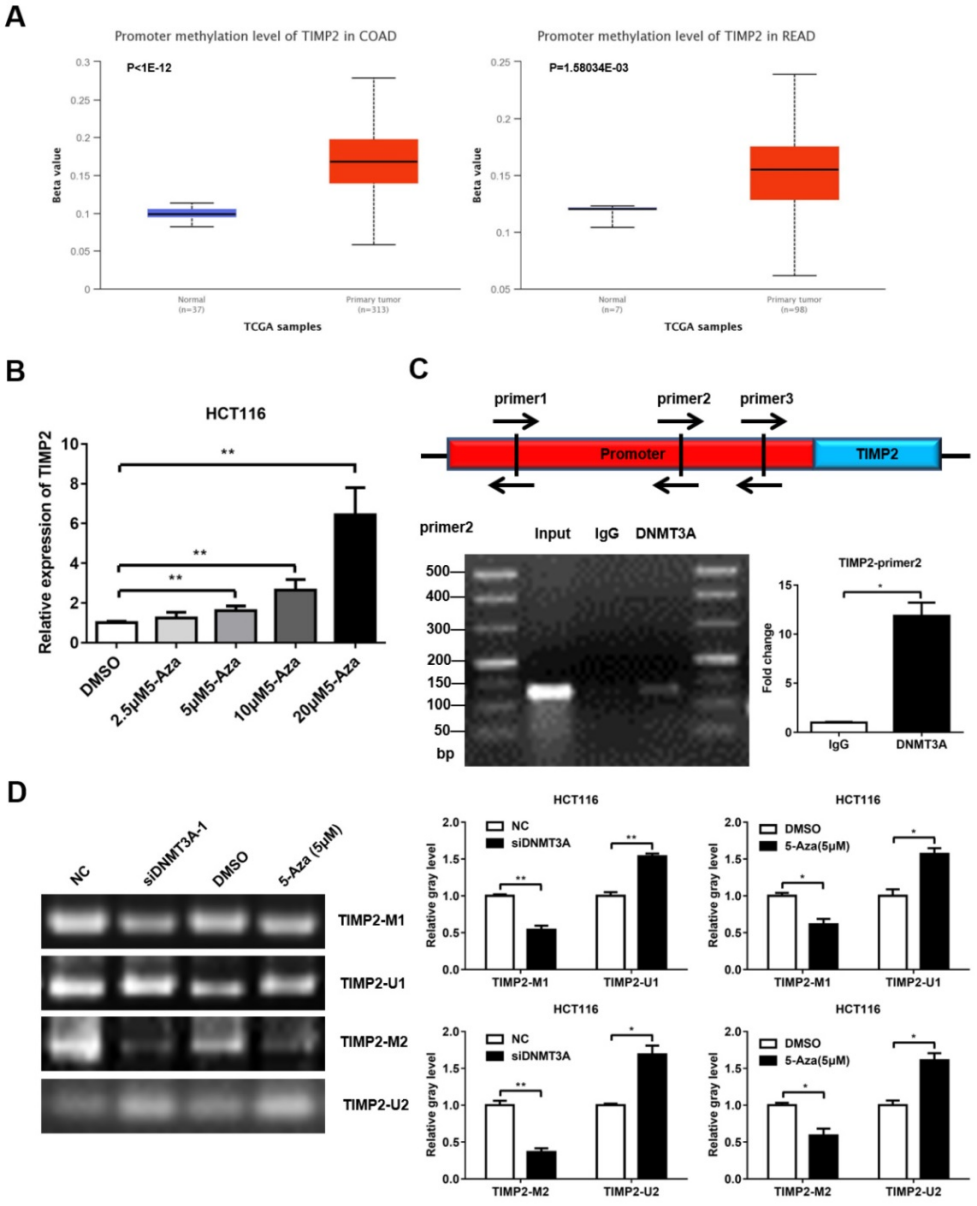
**DNMT3A could regulate the expression of TIMP2. (A)** TIMP2 was hypermethylated in CRC analyzed from UCLCAN database. **(B)** 5-Aza promoted the expression of TIMP2 mRNA. **(C)** ChIP was performed to detected the DNMT3A binding region of TIMP2 promotor.** (D)** MSP was performed to detected the methylation level of TIMP2 promotor. *P<0.05, **P<0.01, ***P<0.001.

DNMT3A belongs to the family of methyltransferases, which regulate gene expression by methylating the CpG islands in the promoter region of the target genes. Previous studies have reported that TIMP2 is hypermethylated in CRC, which may contribute to the high metastasis rate of CRC. We first confirmed that TIMP2 was expressed at low levels in mCRC using western blotting (Supplementary Figure 1A, C). Then, we used the online database UALCAN (http://ualcan.path.uab.edu/) and found that the promoter region of TIMP2 was hypermethylated in the CRC tissue samples (Figure 5A). Next, we treated HCT116 cells with different concentrations of 5-aza-2'-deoxycytidine (5-Aza) for 72 h and detected changes in the TIMP2 mRNA level. As the concentration of 5-Aza increased, the mRNA level of TIMP2 increased significantly, which suggests that TIMP2 mRNA level could be regulated by methylation in human CRC cells (Figure 5B). Combined with our previous data, we assumed that DNMT3A might regulate the transcription level of TIMP2 by methylating the CpG island region in the TIMP2 promoter.

Using online software (http://www.urogene.org/methprimer2/), we analyzed the 2,000 bp DNA sequence upstream of TIMP2, found three potential CpG islands, and designed three pairs of Chip primers for these regions (Figure 5C and Table S1). Then, these were used to perform PCR amplification on the DNA fragments that were precipitated using DNMT3A antibody or IgG from the HCT116 lysate. The PCR products were identified via agarose gel electrophoresis. Among them, the amount of product amplified using the primer pair 2 was significantly higher in the DNMT3A group than in the IgG group (Figure 5C). These data indicate that DNMT3A could bind to the primer pair 2 region of the *TIMP2* promoter. Next, we used MSP to directly verify the methylation effect of DNMT3A on the *TIMP2* promoter. Methylated (TIMP2-M1 and TIMP2-M2) and non-methylated primers (TIMP2-U1 and TIMP2-U2) were designed for the potential CpG islands in the *TIMP2* promoter region. The imaging results showed that, consistent with the results obtained after 5-Aza treatment, the siDNMT3A group showed a significant reduction in the levels of the products amplified using the methylated primers, while the levels of the products amplified using the non-methylated primers significantly increased compared to those of the NC group (Figure 5D). These data show that DNMT3A could directly bind to the *TIMP2* promoter region to change its methylation status, thereby regulating the transcription of *TIMP2* in CRC cells.

### The TDG-DNMT3A-TIMP2 pathway affects colon cancer cell migration and invasion

**Figure 6:**
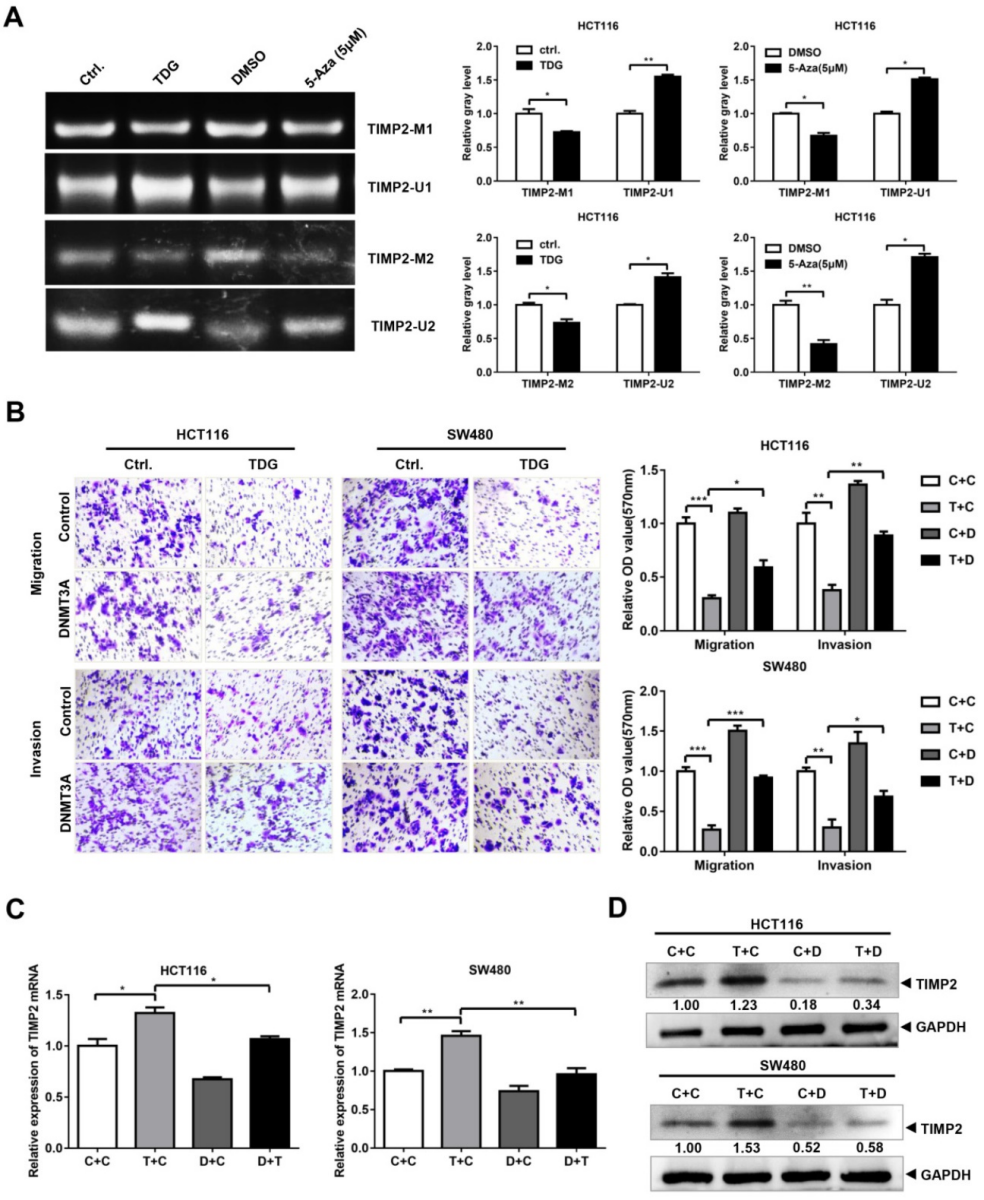
**DNMT3A Partially reversed the effect of TDG in colon cancer cells. (A)** MSP was performed to detected the methylation level of TIMP2 promotor. **(B)** The transwell assay was performed for migration and invasion of colon cancer cells (C+C:Ctrl.+Control, T+C: TDG+Control, C+D: Ctrl.+DNMT3A, T+D: TDG+DNMT3A). **(C-D)** The expression of TIMP2 was detected by qRT-PCR and western blotting. *P<0.05, **P<0.01, ***P<0.001.

Since TDG binds to DNMT3A and promotes its degradation, and DNMT3A can bind to the *TIMP2* promoter to regulate its methylation and *TIMP2* transcription, does TDG also change the methylation of the *TIMP2* promoter and inhibit CRC metastasis through the TDG-DNMT3A-TIMP2 pathway? To answer this question, first, we performed an MSP experiment to examine the TDG-induced methylation of the *TIMP2* promoter. The imaging results showed that compared with the Ctrl. group, the levels of the products amplified using the methylated primers in the TDG overexpression group were significantly reduced, whereas those of the products amplified using the non-methylated primers were significantly increased (Figure 6A). Thus, TDG could induce the hypomethylation of the *TIMP2* promoter, which in turn increases the transcription and expression of *TIMP2* (Figure 2D).

To verify that TDG inhibits the migration and invasion of colon cancer cells through the DNMT3A-TIMP2 axis, we performed a rescue experiment. The TDG and DNMT3A overexpression plasmids were separately co-transfected into HCT116 and SW480 cells. The Transwell assay data showed that the overexpression of DNMT3A partially reversed the decrease in the migration and invasion abilities of colon cancer cells that had been induced by TDG overexpression (Figure 6B). Similarly, the overexpression of DNMT3A partially reversed the increase in the TIMP2 mRNA and protein levels in CRC cells induced by TDG overexpression (Figure 6C). Therefore, TDG promotes the ubiquitination and degradation of DNMT3A by binding to it, decreasing the methylation level of the *TIMP2* promoter, increasing the transcription and expression of TIMP2, and inhibiting the migration and invasion abilities of human CRC cells. Our findings will enrich the knowledge about the regulatory mechanism of TDG in tumors and provide new ideas for the treatment of CRC.

## Discussion

TDG is a mismatch-specific DNA glycosidase that was first identified in HeLa cells. It acts on G/T or G/U mismatches, repairs the bases that have been mismatched with G, and initiates the BER pathway 4. Moreover, TDG can remove new cytosine derivatives produced by ten-eleven translocation enzymes during active DNA demethylation and participates in genome demethylation 11, 12. TDG also interacts with transcription factors or transcription co-activators to regulate gene transcription 13, 14. Considering its roles in DNA repair, DNA demethylation, and transcription regulation, TDG may contribute to carcinogenesis or cancer progression. In multiple myeloma, the TDG expression is decreased, leading to a deficiency in the DNA repair activity in repose to hydrogen peroxide-induced DNA damage 15. In osteosarcomas that arise in *p53* heterozygous mice, the *tdg* expression levels are increased 16. At present, the relationship between BER deficiency and cancer is limited to an autosomal-recessive MAP disease 10, and research on TDG in carcinogenesis and cancer development is limited and needs to be explored in specific cancers.

In our study, we found that the TDG expression was significantly related to metastasis and the prognosis of patients with CRC (Figure 1A-B). Data obtained from clinical CRC samples and human CRC cells also showed a lower TDG expression in the mCRC tissues and human CRC cells (Figure 1C-E). This suggests that TDG plays an important role in the progression of CRC and is worthy of further in-depth study. Data from *in vitro* and *in vivo* overexpression experiments demonstrated that TDG significantly inhibited the migration and invasion of human CRC cells, with an increase in the TIMP2 mRNA and protein levels (Figure 2). Therefore, TDG inhibits metastasis in CRC cells. Yatsuoka et al. detected a low expression of TDG mRNA in pancreatic cancer tissues and cell lines 17. Further, TDG expression was lost due to a heterozygous missense mutation in patients with malignant rectal cancer. TDG inactivation may contribute to the aggressive phenotype of rectal cancer 10. Our findings strongly suggest that TDG might inhibit metastasis in CRC and play a role similar to that of tumor suppressors.

DNMT3A regulates the DNA methylation patterns during mammalian development, and the abnormal DNA methylation patterns induced by DNMT3A can cause many types of cancers 18, 19. Fabbri reported that in lung cancer tissues, miR-29s directly target the 3'-UTR region of DNMT3A and degrade the mRNA of DNMT3A 20. In patients with CRC and acute leukemia, miR-143 negatively regulates the expression of DNMT3A 21, 22. In our study, although TDG did not affect the mRNA level of DNMT3A, it significantly caused a decrease in the protein level of DNMT3A (Figure 3A-B). DNMT3A has been reported to bind to TDG to achieve genome demethylation. Li et al. found that both the PWWP domain and the catalytic domain of DNMT3A could interact with the N-terminus of TDG and affect its enzyme activities 7. In acute myeloid leukemia, TDG interacts with DNMT3A to reduce its methylation ability 23. TDG can bind to the DNMT3A-H3 tail and plays a dominant role in the modulation of DNMT3A activity 24. Using Co-IP and inhibitors, we also found that TDG bound to DNMT3A and promoted its degradation to induce the hypomethylation status of DNMT3A-targeting genes, such as *TIMP2* (Figure 3C-G).

TIMP inhibits the degradation of the extracellular matrix, suppresses angiogenesis, and plays an important role in the occurrence, invasion, and metastasis of tumor cells 25. TIMP2 is reported to be dysregulated in more than 30 clinical studies on breast 26, lung 27, and prostate cancer 28. EZH2 can methylate the promoter of *TIMP2*, thereby inhibiting *TIMP2* expression and promoting metastasis in ovarian cancer 29. Here, we also found that TIMP2 was regulated via methylation in human colon cancer cells (Figure 5A-B). Using the interference of DNMT3A, ChIP, and MSP experiments, we demonstrated that DNMT3A can bind to the *TIMP2* promoter to induce methylation, thereby inhibiting TIMP2 mRNA expression (Figure 5C-E). TDG overexpression reduced the methylation level of the *TIMP2* promoter (Figure 6A) and increased the expression of TIMP2 (Figure 2D-E). DNMT3A overexpression partially rescued the TDG-induced decrease in the migration and invasion abilities of colon cancer cells, and rescued the TDG-induced increase in the TIMP2 expression (Figure 6B-D). The TDG itself has no demethyltransferase activity. Therefore, we propose that TDG can inhibit the migration and invasion abilities of colon cancer cells by reducing their level of DNMT3A, decreasing the *TIMP2* promoter methylation, and increasing the expression of TIMP2. TIMP2 can hydrolyze MMP2 and MMP9 and decrease their protein levels 30, 31. This is consistent with our results (Figure 2D-E).

**Figure 7:**
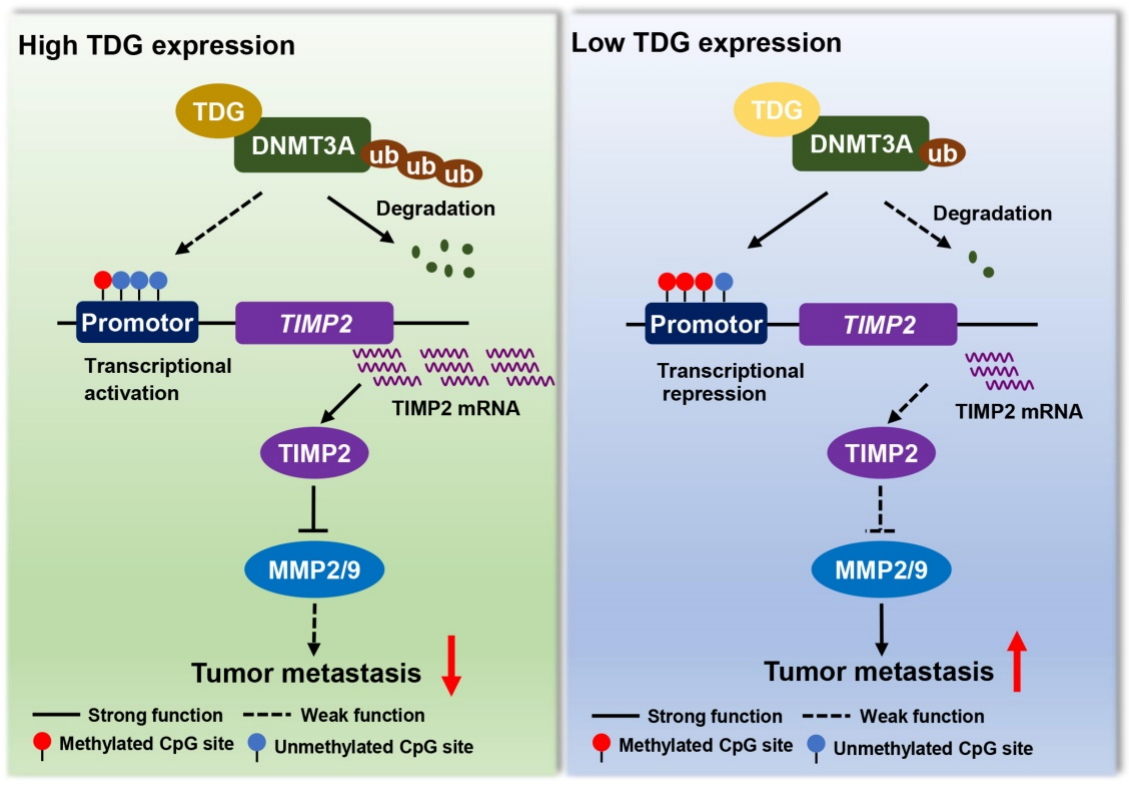
A model for TDG-mediated TIMP2 demethylation in colorectal cancer.

In summary, TDG interacts with DNMT3A to promote ubiquitination degradation, induces the hypomethylation of the *TIMP2* promoter, increases TIMP2 expression, and significantly inhibits the migration and invasion abilities of human colon cancer cells (Figure 7). This epigenetic regulatory role of TDG in inhibiting the metastasis of CRC enriches the existing knowledge on the regulatory mechanisms of TDG and the therapeutic strategies that may be used for CRC.

## Supplementary Material

Supplementary figure and table.

## Abbreviations

TDG: Thymine DNA glycosylase
DNMT3A: DNA methyltransferase 3 alpha
CRC: Colorectal cancer
Co-IP: Co-immunoprecipitation
CHIP: Chromatin immunoprecipitation analysis
MSP: Methylation-specific PCR
TIMP2: TIMP metallopeptidase inhibitor 2
TCGA: The Cancer Genome Atlas
