# Supplementary material for: TDG suppresses the migration and invasion of human colon cancer cells via the DNMT3A/TIMP2 axis
Source: Int J Biol Sci. 2022 Mar 21;18(6):2527–39. doi: 10.7150/ijbs.69266 (PMC8990457; doi:10.7150/ijbs.69266)
Supplement: Supplementary file 1 — Supplementary figure and table. [file ijbsv18p2527s1.pdf]

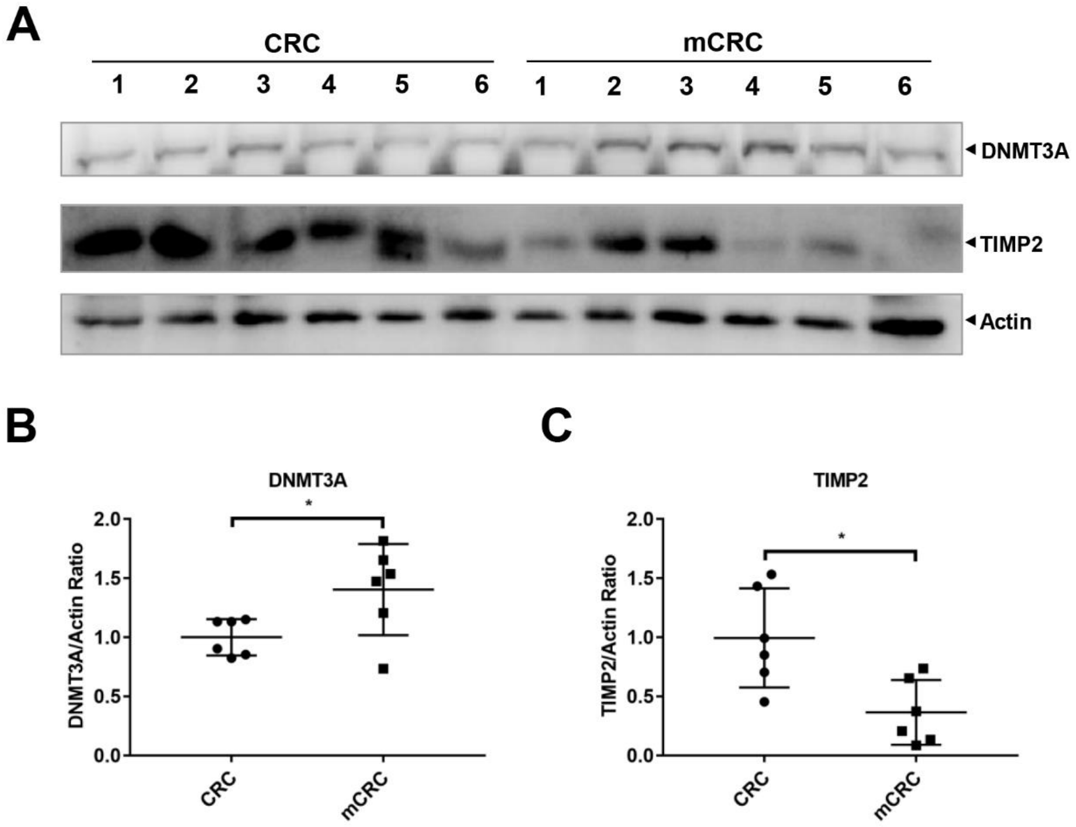

Supplementary Figure 1. The protein level of DNMT3A and TIMP2 in CRC and mCRC patient tissues. (A) Detection of DNMT3A and TIMP2 by western blotting. **(B)** Quantification of DNMT3A protein. **(C)** Quantification of TIMP2 protein. *P<0.05

Table S1：Primers and oligonucleotides used in this study

Name	Sequence (5'-3')
GAPDH-Forward	CAATGACCCCTTCATTGACC
GAPDH Reverse	GACAAGCTTCCCGTTCTCAG
TDG-Forward	GGCTAATTGAGAGCGTGGAG
TDG-Reverse	GCATGGCTTTCTTCTTCCTG
MMP2-Forward	CTTCTTCCCTCGCAAGCC
MMP-Reverse	ATGGATTCGAGAAAACCG
MMP9-Forward	ACGCAGACATCGTCATCC
MMP9-Reverse	AACCGAGTTGAACCACG
TIMP1-Forward	GCAATTCCGACCTCGTCATCA
TIMP1-Reverse	GCAATTCCGACCTCGTCATCA
TIMP2-Forward	CAGATGTAGTGATCAGGGCCA
TIMP2-Reverse	AGGGCACGATGAAGTCACAG
DNMT3A-Forward	TATTGATGAGGCGCACAAGAGAGC
DNMT3A-Reverse	GGGTGTTCCAGGGTAACATTGAG
NC	UUCUCCGAACGUGUCACGUTT
siDNMT3A-1	GCCUCAAGAGCAGUGGAAATT
siDNMT3A-2	GGUCAUUGCAGGAAUGAAUTT
TIMP2primer 1-Forward	GCCCGGCTAATTTCTTGTG
TIMP2primer 1-Reverse	GCCTGTAATCCCAGCACTTT
TIMP2primer 2-Forward	GGAGGTGGCGACAGGGAA
TIMP2primer 2-Reverse	CAGCTCGACTCTGGAGGGCTA
TIMP2primer 3-Forward	CGGGAGGAGGAGCAGAAA
TIMP2primer 3-Reverse	CGCTGCCTTCTACGGATGT
TIMP2-MF1	TAGGTTGGAGTGTAGTGGCGTA
TIMP2-MR1	TTCGAAACCAACCTAACGACT
TIMP2-UF1	TAGGTTGGAGTGTAGTGGTGTAA
TIMP2-UR1	AAAAATTCAAAACCAACCTAACAACT
TIMP2-MF2	TTTGGTGTTTTGGAAGAACGGGCG
TIMP2-MR2	CGACCCCGATCCCCGCTACG
TIMP2-UF2	TTTGGTGTTTTGGAAGAATGGGTC
TIMP2-UR2	CCAACCCCAATCCCCACTACA
